# The integrated stress response in budding yeast lifespan extension

**DOI:** 10.15698/mic2017.11.597

**Published:** 2017-10-24

**Authors:** Spike D.L. Postnikoff, Jay E. Johnson, Jessica K. Tyler

**Affiliations:** 1 Department of Pathology and Laboratory Medicine, Weill Cornell Medicine, 1300 York Avenue, New York, NY 10065.; 2 Orentreich Foundation for the Advancement of Science, Cold Spring, NY.

**Keywords:** replicative lifespan, chronological lifespan, budding yeast, integrated stress response, autophagy

## Abstract

Aging is a complex, multi-factorial biological process shared by all living organisms. It is manifested by a gradual accumulation of molecular alterations that lead to the decline of normal physiological functions in a time-dependent fashion. The ultimate goal of aging research is to develop therapeutic means to extend human lifespan, while reducing susceptibility to many age-related diseases including cancer, as well as metabolic, cardiovascular and neurodegenerative disorders. However, this first requires elucidation of the causes of aging, which has been greatly facilitated by the use of model organisms. In particular, the budding yeast Saccharomyces cerevisiae has been invaluable in the identification of conserved molecular and cellular determinants of aging and for the development of approaches to manipulate these aging determinants to extend lifespan. Strikingly, where examined, virtually all means to experimentally extend lifespan result in the induction of cellular stress responses. This review describes growing evidence in yeast that activation of the integrated stress response contributes significantly to lifespan extension. These findings demonstrate that yeast remains a powerful model system for elucidating conserved mechanisms to achieve lifespan extension that are likely to drive therapeutic approaches to extend human lifespan and healthspan.

## INTRODUCTION TO YEAST AGING

The intent of lifespan-extending regimens is to prevent and treat the major diseases of society. However aging studies in humans and mammals are hampered by their very long lifespan. Thus, short-lived organisms have gained popularity as experimental models for aging studies. In particular, budding yeast is a highly informative model for aging studies with its short lifespan, genetic tools, and fully sequenced genome 1,2. Though unicellular, yeast has been a remarkable experimental model to identify and characterize evolutionarily conserved basic biological processes, including aging. Accordingly, research in budding yeast continues to uncover the conserved pathways that cause aging in all eukaryotes 3, leading to the identification of genes and interventions that can mediate lifespan extension. For example, potential anti-aging drugs such as rapamycin, resveratrol, and spermidine, and promising longevity factors (e.g. Sir2, Tor1, Sch9, Ras) were first identified and characterized in yeast before they were adopted and confirmed to work in metazoans 4.

Budding yeast provides two separate, but partially overlapping paradigms for aging studies, replicative lifespan (RLS) and chronological lifespan (CLS) 4. All eukaryotic cells undergo a finite number of cell divisions, before irreversibly exiting mitosis. RLS measures the number of daughter cells produced by a mother cell before senescence and is a model for studying aging of mitotically active cells. CLS determines the time that cells survive in a non-dividing state after depletion of nutrients in stationary phase and is a model for the study of post-mitotic cells. The fact that chronologically old yeast also have a shorter RLS 5,6 indicates that RLS and CLS share core mechanisms.

## INTRODUCTION TO THE YEAST INTEGRATED STRESS RESPONSE

Strikingly, most interventions that extend lifespan are, or induce, limited amounts of stress that have a beneficial effect via the phenomenon of hormesis, because these stresses would be toxic or lethal at higher doses. These lifespan-extending hormetic stresses induce a protective cellular stress response. This conserved stress response in yeast was termed the General Amino Acid Control (GAAC) because it was initially identified as a response to amino acid depletion that upregulates the genes required for amino acid synthesis 7. However, we now know that this same yeast stress response pathway is also activated by starvation for purines 8, glucose limitation/depletion 9, ER stress 10, growth on non-fermentable carbon sources like ethanol 9, high salt 11, treatment with alkylating agents 12, and TOR inhibition 13,14. As such, here we refer to this yeast stress response pathway by the name used in multicelluar eukaryotes, the Integrated Stress Response (ISR) 15. As discussed below, multiple different lifespan-extending regimens require the ISR for lifespan extension. As the ISR has been reviewed extensively recently 16, here we focus on the contribution of the yeast ISR to lifespan extension. Other stress responses, such as the retrograde response, have also been implicated in the extension of CLS and RLS of yeast, and have been extensively reviewed 17,18, so will not be discussed here.

**Figure 1:**
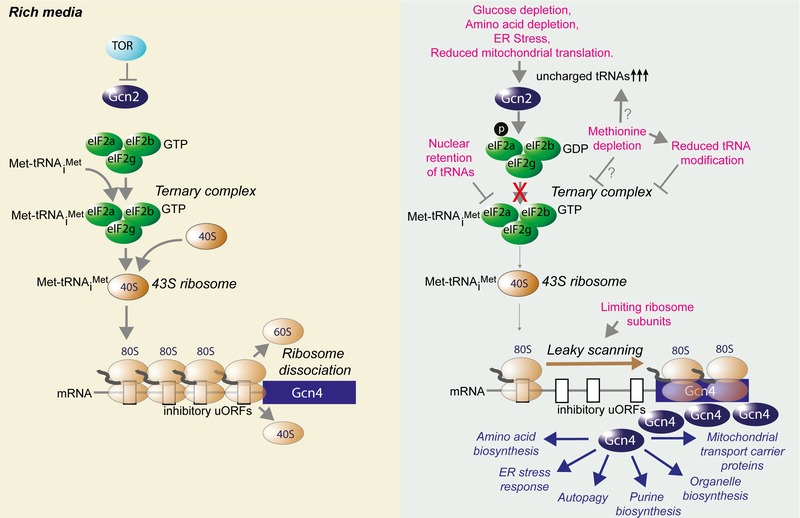
FIGURE 1: Overview of the yeast integrated stress response and how it is influenced by lifespan extending interventions. During growth in nutrient rich conditions, the ternary complex is formed from eIF2, methionine bound to tRNA_i_^Met^, and GTP, which then delivers Met-tRNA_i_^Met^ to the 40S ribosome to make the 43S complex, which is a key rate-limiting step in translational initiation. Under rich growth conditions, efficient translation of the inhibitory upstream ORFs (uORFs) of the *GCN4* mRNA causes dissociation of the ribosome, preventing it scanning along the mRNA to the protein coding ORF (blue). The regimens indicated in pink, which all extend RLS, either result in increased abundance of uncharged tRNAs that activate Gcn2 to phosphorylate eIF2α to prevent formation of functional eIF2-GTP, or they activate Gcn4 in a Gcn2-independent manner, in conditions that reduce formation of the ternary complex directly (reduced amounts of processed tRNAs in the cytoplasm, reduced amino acids) or via reduced amounts of ribosomal subunits. In either case, the end result is inefficient 80S ribosome formation, which promotes leaky scanning along the mRNA to the protein coding ORF of *GCN4.* The resulting Gcn4 protein induces transcription of genes required for the indicated stress response pathways.

During the ISR in yeast, a variety of stressful conditions activate the kinase Gcn2, which phosphorylates eIF2α, the central regulation node of the ISR. Phosphorylation of eIF2α promotes a stress-resistant state by global attenuation of protein synthesis, which reduces the load of unfolded proteins on chaperone networks and diverts amino acids from energetically costly protein synthesis to other metabolic pathways. Although the mammalian ISR has three other eIF2α kinases in addition to Gcn2, the stress response pathways are otherwise very similar in yeast and mammals 16. Specifically, eIF2α phosphorylation downregulates formation of the eIF2 complex, resulting in reduced formation of the ternary complex containing eIF2, GTP and the initiator Methionyl-tRNA (Met-tRNA_i_^Met^). This in turn reduces delivery of Met-tRNA_i_^Met^ to the 40S ribosome, hampering initiation of global cap-dependent translation 19 (Fig. 1). In addition to these general effects on protein and amino acid metabolism, eIF2α phosphorylation also serves to activate a gene expression program that depends on translational upregulation of Gcn4. The mechanism for the paradoxical synthesis of the ISR effector protein Gcn4 in the face of global translational downregulation has been revealed by the elegant work of the Hinnebusch lab 19. Under normal conditions, translation of the short upstream open reading frames (uORFs) within the *GCN4* mRNA leader occurs, followed by the ribosomes dissociating from the *GCN4* mRNA, precluding initiation at the *GCN4* start codon (Fig. 1). However, under conditions where there are limiting amounts of 40S ribosome associated with Met-tRNA_i_^Met^, the ribosomes scan through the inhibitory uORFs within the *GCN4* leader and initiate translation at the *GCN4* start codon. The mammalian counterpart of Gcn4, ATF4, is induced by an analogous mode of translational control 20.

Gcn4 can also be synthesized in the absence of Gcn2 activation, by conditions that directly reduce global cap-dependent translation (Fig. 1). For example, Gcn4 is translated in yeast strains lacking Gcn2 upon rapid depletion of amino acids 21, which presumably limits the formation of the ternary complex. Mutants that limit tRNA processing or tRNA nuclear export, such as *los1*∆*,* also activate Gcn4 in the absence of Gcn2, presumably because there is insufficient amount of ternary complex formation 22,23. Similarly, reduced abundance of large ribosomal subunits could also trigger Gcn4 translation without the need for Gcn2 24 as there would be less 60S ribosome to associate with the 40S-Met-tRNA_i_^Met^ complex. Notably, amino acid depletion is a clear mechanism to extend both RLS and CLS, while reduced abundance of ribosomal subunits or deletion of the *LOS1* gene required for tRNA export extend RLS in a manner dependent on Gcn4 but not Gcn2 22,23,24.

The Gcn4 transcriptional activator upregulates genes that allow adaptation to cellular stress 16. These include genes that mediate amino acid biosynthesis, purine biosynthesis, organelle biosynthesis, ER stress response, mitochondrial carrier proteins and autophagy 16 (Fig. 1). Although multiple stresses converge on eIF2α phosphorylation, the cellular outcome is not always the same. The effect of ISR activation depends on the nature of the stress, its duration, and the amount of stress, which in turn affects the extent of eIF2α phosphorylation and Gcn4 synthesis. A short-lived ISR is usually an adaptive response aimed at restoring homeostasis, while prolonged ISR activation can signal for cell death.

We are still at an early stage in trying to understand which of the downstream stress response pathways induced by Gcn4 are key to the lifespan extension mediated by the ISR (Fig. 1). Although not conclusive, some clues as to the relative importance of the different ISR-induced stress response pathways to lifespan can be provided by the phenotypes resulting from deletion of genes activated by the ISR. For example, it is unlikely that purine biosynthesis is key for lifespan extension, given that deletion of purine biosynthesis genes extends CLS 25. Similarly, RLS is extended by deletion of various genes involved in amino acid synthesis 23 while deletion of multiple unfolded protein response (UPR) target genes extends RLS 26. Furthermore, mitochondrial carrier proteins are degraded via a novel selective autophagy pathway in replicatively-aged yeast 27, warranting investigation into their role in lifespan determination. As such, the beneficial effects of inactivation of key genes in these ISR-target pathways are not consistent with their transcriptional induction extending lifespan. In further support of such an idea, the UPR is not even required for the lifespan extension that occurs in response to ER stress 28, as discussed in more detail below. By default, that leaves autophagy as the most promising target of the ISR for lifespan extension. By coincidence, autophagy has become of particular interest to the aging field 29,30.

Autophagy is a conserved catabolic housekeeping mechanism whereby bulk cytoplasm, including proteins and organelles, are sequestered into membrane-bound vesicles (autophagosomes) which subsequently fuse with vacuoles where their content is degraded by vacuolar proteases 31. The identification of the autophagy genes and the mechanism of autophagy, like many key processes, occurred in yeast, in this case leading to the 2016 Nobel prizes in Physiology or Medicine 32. Many of the genes and mechanisms are highly conserved in metazoans, including humans, with abnormalities in higher eukaryote autophagy resulting in the development of aging and age-related diseases, as previously reviewed 29,33. Elsewhere, we have recently reviewed the contributions of autophagy to yeast lifespan extension 34. Here, we synthesize the recent studies that link the ISR to lifespan extension in yeast, to support our proposal that the mechanism whereby stress extends lifespan is via induction of the ISR and downstream stress response pathways, including autophagy. By extension, we suggest that therapeutics that specifically induce the ISR and/or autophagy should be developed to promote human healthspan and extend lifespan.

## THE ROLE OF THE ISR IN YEAST LIFESPAN EXTENSION

### Nutrient depletion extends RLS and activates the ISR

The yeast RLS assay utilizes the asymmetric division of mother and daughter yeast cells to track the replicative capacity of individual cells, and has been used to identify various types of factors that contribute to replicative senescence. Comparison of daughter cells and old mother cells has revealed that many factors selectively accumulate in the mother cells, and these so-called senescence factors have been suggested to drive the aging process. These senescence factors include extra-ribosomal DNA circles 35, damaged nuclear pore complexes 36, cytosolic aggregates, oxidized proteins 37, and dysfunctional mitochondria 38. In essence, mechanisms exist to exclude the senescence factors out of the daughter cells, to enable them to be rejuvenated while at the same time, causing the mother cells to age. In theory, any condition that results in the activation of autophagy to remove accumulating senescence factors, including damaged or aggregated proteins and damaged organelles and to recycle cellular components, would be a logical approach to extend the RLS of mother cells. Where examined, this is transpiring to be the case.

There is currently no evidence to suggest that either Gcn4 or autophagy contribute to longevity under nutrient-rich growth conditions. This is consistent with the fact that deletion of most *ATG* genes or *GCN4* has minimal effects on RLS 23,28. However, many of the interventions that extend yeast RLS also induce the ISR and/or autophagy. We propose that these RLS extending regimens extend lifespan via inducing the ISR and/or autophagy, as discussed below. Notably, many of the therapeutic, genetic or environmental interventions that extend yeast RLS are either a decrease of nutrients per se or they decrease the activity of the nutrient signaling pathways. Accordingly, inactivation of either of the two major nutrient signaling pathways extends yeast RLS: the target of rapamycin (TOR) / serine-threonine kinase Sch9 pathway 39 and the RAS / cAMP-dependent protein kinase A (PKA) pathway 40. Exactly how these pathways regulate RLS is unclear, but in general, when nutrients are abundant, these nutrient sensing pathways inhibit the ISR and autophagy (Fig. 1). However, when nutrients are limiting, the nutrient sensing pathways are inactivated, their inhibitory influence on autophagy and the ISR is released, and yeast RLS is extended. So far, the relationship between RLS extension and autophagy induction upon inactivation of the nutrient sensing pathways is purely correlation. It has not yet been tested whether the induction of autophagy upon inhibition of the TOR/Sch9 or RAS/cAMP pathways is required for RLS extension, or whether there is any involvement of the ISR in their ability to extend RLS.

### The role of the yeast ISR in autophagy

Gcn2 and Gcn4 were initially described as being required for the induction of autophagy upon nitrogen starvation. Specifically, deletion of *GCN2* or *GCN4* blocked nitrogen starvation-induced macroautophagy, as determined by electron micrography of large autophagosomes 41. Closer examination in a later study uncovered that in response to nitrogen depletion, deletion of *GCN2* or *GCN4* only partially diminished autophagic flux, but formed multiple small autophagosomes, rather than large autophagosomes 42. This phenotype was due to failure to transcriptionally upregulate Atg8 levels in the absence of Gcn2/Gcn4. In agreement with the fact that Gcn4 is induced at the translational level, the signaling mechanism that induces the initiation of macroautophagy occurs independent of new protein synthesis, while protein synthesis is required for the formation of large autophagosomes 43. Autophagosome size depends on the transcriptional upregulation of Atg8 44, which requires Gcn4 42. As such, Gcn2/Gcn4 are required for enlargement of autophagosomes, but not for autophagy per se upon nitrogen starvation. Gcn4 is also required for *ATG1* and *ATG32* transcription upon nitrogen starvation 45, where Atg1 is required for the initiation of autophagy and Atg32 is specifically required for mitophagy, the form of autophagy that selectively degrades mitochondria. Furthermore, many *ATG* genes have Gcn4 binding sites in their promoters 45. Although nitrogen starvation is the conventional method to induce autophagy, its relevance to RLS is negligible, given that nitrogen starvation induces cell cycle arrest.

Amino acid depletion is another common mechanism used to induce autophagy. Notably, amino acid depletion is not only a mechanism to extend yeast RLS 46,47, but Gcn2/Gcn4 are also essential for macroautophagy upon amino acid depletion 42. Amino acid starvation-induced autophagy responded to the levels of intracellular, not extracellular, amino acids, and Gcn2/Gcn4 were specifically found to be required for autophagic trafficking in response to amino acid starvation 42. The reason why amino acid depletion-triggered autophagy, but not nitrogen starvation-triggered autophagy, absolutely requires Gcn2/Gcn4 is unclear as yet.

### The requirement for the Gcn4 activator in many regimens of RLS extension

Although Gcn2 is conventionally activated by tRNAs that are not bound to amino acids (uncharged tRNAs), Gcn4 synthesis is induced under many conditions besides amino acid depletion. These include starvation for purines 8, glucose limitation 9, ER stress 10, growth on non-fermentable carbon sources like ethanol 9, high salt 11, treatment with alkylating agents 12 and TOR inhibition 13,14. All these conditions that activate Gcn4 depend on the Gcn2 kinase and many extend yeast RLS. The mechanism whereby glucose limitation activates Gcn2 appears to be partially due to the depletion of cytoplasmic pools of amino acids that occurs upon glucose depletion. Accordingly, supplementation with amino acids reduces the Gcn4 synthesis that results from glucose limitation 9. However, the effect is only partial, suggesting that mechanisms of Gcn2 activation that do not rely on uncharged tRNAs may also be at play.

The TOR pathway and the ISR pathway appear to be interlinked, although this is somewhat controversial. One report showed that the activation of autophagy that is triggered by rapamycin-mediated inhibition of TOR was not affected by deletion of *GCN2* 41. By contrast, RLS extension observed upon *TOR1* deletion was mediated at least partially by Gcn4 24, because it was reduced upon *GCN4* deletion. The reason for the discrepancy between the two studies could reflect that, although Gcn2 is activated upon TOR inhibition, Gcn2 activation may not be essential for Gcn4 translation upon TOR inhibition. The reasoning for this is that there could be reduced translation of the uORFs in the *GCN4* leader upon TOR inhibition or *TOR1* deletion as a consequence of loss of TOR-mediated phosphorylation of the eukaryotic initiation factor eIF4E binding protein 4E-BP1 and accompanying down regulation of global protein synthesis. Alternatively, this could be due to potential changes in the small ribosomal subunit caused by decreased phosphorylation of ribosomal protein S6 (RpS6) by Sch9/S6K, which might interfere with 40S structure, changing its activity and increasing the leakiness of translation on the *GCN4* mRNA. As discussed above, Gcn4 can be activated in the absence of Gcn2, in conditions where protein synthesis is reduced (Fig. 1). There is also feedback between the two pathways at least in one direction because inhibition of TOR signaling induces activation of Gcn2 13,14 (Fig. 1). As such, it would appear that inhibition of the TOR pathway stimulates autophagy by not only releasing the inhibitory phosphorylation on Atg13 48,49, but also by increasing Gcn4 synthesis to transcriptionally upregulate *ATG* genes (Fig. 1). Whether Gcn4 synthesis per se is sufficient to extend RLS or induce autophagy remains untested.

Disruptions of the ER-Golgi apparatus can extend RLS by inducing autophagy. In fact, this provides the most compelling evidence to date for a role for autophagy in RLS extension. An innovative genetic screen for deletion mutants that maintain the transcriptional silencing that is usually lost in old cells identified novel gene deletions that extend RLS 28. One of these, *RER1* encodes a receptor that maintains ER compartmentalization, and upon deletion extended RLS by 64% 28. Deletion of *RER1* resulted in ER stress, as it activated the UPR and autophagy, which are likely to mitigate an increase in ER load. In agreement with lifespan extension being caused by ER stress, the drug tunicamycin, which also activates ER stress, extends RLS. The autophagy induced by *RER1* deletion was required for RLS extension, because deletion of genes encoding Atg8, or Atg5, the latter of which mediates the Atg8 lipidation required for expansion of autophagosomes, abolished the otherwise extended RLS in *rer1*∆ yeast. However, the UPR induced by ER stress was not required for RLS extension, because deletion of the *IRE1* gene, encoding the principle sensor of the UPR, did not block RLS extension. The screen also recovered *MNT3*, encoding a Golgi glycosylase as being long-lived upon its deletion. Deletion of *MNT3* led to high basal levels of UPR, elevated autophagy and extended mitotic lifespan 28. As such, induction of autophagy and the accompanying increase in RLS appears to be a broad adaptive response evoked by impaired protein modification or trafficking across the ER-Golgi network. Given that ER stress is known to induce Gcn4 10, it is likely that the lifespan-extending autophagy that is induced by ER stress is dependent on Gcn4.

Polyamines, such as spermidine, play a role in longevity. Polyamine levels, including spermidine, decrease during aging. Notably, addition of spermidine extends yeast RLS when given to old cells, but not young cells, isolated by elutriation 50. Spermidine treatment increases autophagic flux, which may be related to the fact that spermidine leads to transcriptional upregulation of *ATG7*, *ATG11*, and *ATG15*, potentially through its influence on histone acetylation 50. In agreement with the observed induction of autophagic flux, spermidine treatment promotes the removal of oxidized prions from yeast 51. It is not currently known whether the extension of RLS by spermidine is mediated via inducing autophagy, although this appears to be the case with CLS 50. Interestingly, the ability of spermidine to induce transcription of other stress-induced genes, such as the gene encoding the drug:H^+^ antiporter Qdr3, is dependent on the transcription factor Gcn4 52. It is tempting to speculate that spermidine-mediated yeast RLS extension also requires Gcn4, and potentially, Gcn4-dependent autophagy.

Gcn4 is required for RLS extension that occurs upon nuclear retention of tRNAs. Deletion of the gene encoding the tRNA exporter Los1 was found to extend RLS in a systematic analysis of 4,698 single gene deletions for the effect on the yeast RLS 23. The lifespan extension upon *LOS1 *deletion was shown to be in the same pathway as lifespan extension by dietary restriction (DR) via epistasis analyses 23, suggesting that nuclear retention of tRNAs may be one of the mechanisms by which DR extends RLS. Deletion of *LOS1* leads to the activation of Gcn4, reflected in the upregulation of many of its transcriptional targets, and seemingly in the absence of Gcn2 activation, because no global decrease in mRNA translation occurs 23. The limited availability of tRNAs in the cytoplasm would presumably reduce formation of Met-tRNA_i_^Met^ which would promote scanning through the Gcn4 upstream ORFs and Gcn4 synthesis (Fig. 1) without the need for Gcn2 activation. It will be interesting to discern if RLS extension upon *LOS1* deletion is dependent on autophagy or other pathways induced by Gcn4.

Gcn4 is required for RLS extension that results from deletion of specific mitochondrial components. Yeast lacking the gene encoding the mitochondrial AAA protease Afg3 are long-lived 53. The Afg3 protease has multiple functions, including helping to mature a mitochondrial ribosomal protein. This is reminiscent of RLS extension observed in yeast lacking the mitochondrial ribosomal protein Afo1 54. Furthermore, downregulation of mitochondrial protein synthesis by erythromycin extends RLS 55. Taken together, these studies suggest that reduced mitochondrial translation can extend RLS; and in the case of Afg3, RLS extension was dependent on Gcn4, and *AFG3* deletion reduced cellular mRNA translation 53. It is possible that reduced mitochondrial translation activates the ISR, which results in reduced mRNA translation and extended RLS.

### Requirement for the ISR in extension of CLS 

Investigations into the involvement of Gcn2/Gcn4 in yeast CLS extension to date are limited, but are consistent with a role for the ISR in CLS extension. The Chinese herbal compound cryptotanshione extends CLS, but only when there are ample essential amino acids, and this is seemingly through the amino acid sensing pathways 56. Specifically, cryptotanshione inactivates the Tor/Sch9 pathway, leading to elevated Gcn2 activity via release from Tor/Sch9-dependent inhibition (Fig. 1). Demonstrating the importance of these pathways for CLS extension by cryptotanshione, deletion of *TOR1, SCH9* or *GCN2* blocked cryptotanshione from extending CLS 56. Methionine restriction extends organismal lifespan across many different species of eukaryotes 57. Limiting amounts of methionine in synthetic defined media extends yeast CLS in a manner dependent on *GCN2*, but independent of *TOR1* 58. The mechanism at play appears to be due to increased levels of uncharged tRNAs that result from methionine depletion 59, activating Gcn2 and the ISR (Fig. 1). Addition, not depletion, of other specific amino acids to minimal media has been shown to extend CLS, and deletion of *GCN4* blocks this CLS extension 60. As such, there are clear examples of the ISR being required for CLS extension. Whether these mechanisms are through autophagy or other downstream responses of the ISR is unknown at present.

## SUMMARY AND REMAINING QUESTIONS

Yeast aging models have been indispensable for elucidating mechanisms of cellular aging, especially with regards to nutrient signaling pathways. During an organism’s lifetime, aging cells undergo a multitude of changes and accumulate macromolecular damage. Decrease in high-nutrient PKA/Sch9/TOR signaling pathways leads to a slowing of the aging processes, likely due to the increased stress resistance and the regulation of proteostatic mechanisms. In this review we provide an overview of emerging evidence indicating the importance of the ISR as a parallel, yet opposing, nutrient-signaling mechanism to TOR signaling. Also, we highlight findings suggesting a fundamental role of the ISR as a target effector of nutrient signaling on yeast lifespan.

Although growing evidence suggests that the ISR is required for lifespan extension, we do not yet know if the ISR is sufficient for lifespan extension. Furthermore, is Gcn4 activation sufficient to extend yeast RLS or CLS? If this were the case, it would suggest that the general inhibition of cap-dependent translation is not required for yeast lifespan extension: rather it is the resulting upregulation of stress response gene expression that is key. As such, increasing Gcn4/ATF4 protein levels may be a promising therapeutic intervention to extend lifespan and healthspan without the detrimental effects of reduced global protein synthesis and immunosuppression that occur in humans upon TOR inhibition by compounds such as rapamycin. Notably, it is not yet clear whether the autophagy that is induced by lifespan extension regimens is dependent on Gcn4, or other autophagy-inducing pathways. Thus, therapeutics to directly activate autophagy could be another promising approach to extend lifespan and healthspan.

Gcn4 induces transcription of limiting components of multiple different stress response pathways (Fig. 1). This raises the question: is autophagy the only downstream process activated by Gcn4 that is required for lifespan extension? This could be answered by testing whether induction of autophagy is sufficient to extend yeast lifespan. This is clearly the case in multicellular eukaryotes because overexpression of Atg5 in mice, which increases autophagic flux, is sufficient to extend lifespan 61. There are many different types of autophagy, and a better understanding of the aging process would be gained by knowledge of which specific autophagy pathways need to be activated to extend yeast lifespan.

The phenomenal pace of research and discoveries being made using the yeast model organism indicate that it will continue to be a central model for aging studies for the foreseeable future. Critically, since many of the components of longevity-regulating mechanisms are highly conserved from yeast to higher eukaryotes, the discovery of pharmaceutical and physiological regulators of these processes in yeast will likely have biological significance to human health and aging.

## References

[1] Bitterman KJ, Medvedik O, Sinclair DA (2003). Longevity regulation in Saccharomyces cerevisiae: linking metabolism, genome stability, and heterochromatin.. Microbiol Mol Biol Rev.

[2] Steinkraus KA, Kaeberlein M, Kennedy BK (2008). Replicative aging in yeast: the means to the end.. Annu Rev Cell Dev Biol.

[3] Janssens GE, Veenhoff LM (2016). Evidence for the hallmarks of human aging in replicatively aging yeast.. Microb Cell.

[4] Longo VD, Shadel GS, Kaeberlein M, Kennedy B (2012). Replicative and chronological aging in Saccharomyces cerevisiae.. Cell Metab.

[5] Burtner CR, Murakami CJ, Kennedy BK, Kaeberlein M (2009). A molecular mechanism of chronological aging in yeast.. Cell Cycle.

[6] Fabrizio P, Battistella L, Vardavas R, Gattazzo C, Liou LL, Diaspro A, Dossen JW, Gralla EB, Longo VD (2004). Superoxide is a mediator of an altruistic aging program in Saccharomyces cerevisiae.. J Cell Biol.

[7] Hinnebusch AG, Fink GR (1983). Positive regulation in the general amino acid control of Saccharomyces cerevisiae.. Proc Natl Acad Sci U S A.

[8] Rolfes RJ, Hinnebusch AG (1993). Translation of the yeast transcriptional activator GCN4 is stimulated by purine limitation: implications for activation of the protein kinase GCN2.. Mol Cell Biol.

[9] Yang R, Wek SA, Wek RC (2000). Glucose limitation induces GCN4 translation by activation of Gcn2 protein kinase.. Mol Cell Biol.

[10] Deloche O, de la Cruz J, Kressler D, Doere M, Linder P (2004). A membrane transport defect leads to a rapid attenuation of translation initiation in Saccharomyces cerevisiae.. Mol Cell.

[11] Goossens A, Dever TE, Pascual-Ahuir A, Serrano R (2001). The protein kinase Gcn2p mediates sodium toxicity in yeast.. J Biol Chem.

[12] Natarajan K, Meyer MR, Jackson BM, Slade D, Roberts C, Hinnebusch AG, Marton MJ (2001). Transcriptional profiling shows that Gcn4p is a master regulator of gene expression during amino acid starvation in yeast.. Mol Cell Biol.

[13] Cherkasova VA, Hinnebusch AG (2003). Translational control by TOR and TAP42 through dephosphorylation of eIF2alpha kinase GCN2.. Genes Dev.

[14] Kubota H, Obata T, Ota K, Sasaki T, Ito T (2003). Rapamycin-induced translational derepression of GCN4 mRNA involves a novel mechanism for activation of the eIF2 alpha kinase GCN2.. J Biol Chem.

[15] Harding HP, Zhang Y, Zeng H, Novoa I, Lu PD, Calfon M, Sadri N, Yun C, Popko B, Paules R, Stojdl DF, Bell JC, Hettmann T, Leiden JM, Ron D (2003). An integrated stress response regulates amino acid metabolism and resistance to oxidative stress.. Mol Cell.

[16] Pakos-Zebrucka K, Koryga I, Mnich K, Ljujic M, Samali A, Gorman AM (2016). The integrated stress response.. EMBO Rep.

[17] Quiros PM, Mottis A, Auwerx J (2016). Mitonuclear communication in homeostasis and stress.. Nat Rev Mol Cell Biol.

[18] Jazwinski SM (2014). The retrograde response: a conserved compensatory reaction to damage from within and from without.. Prog Mol Biol Transl Sci.

[19] Hinnebusch AG (2005). Translational regulation of GCN4 and the general amino acid control of yeast.. Annu Rev Microbiol.

[20] Jackson RJ, Hellen CU, Pestova TV (2010). The mechanism of eukaryotic translation initiation and principles of its regulation.. Nat Rev Mol Cell Biol.

[21] Tzamarias D, Roussou I, Thireos G (1989). Coupling of GCN4 mRNA translational activation with decreased rates of polypeptide chain initiation.. Cell.

[22] Qiu H, Hu C, Anderson J, Bjork GR, Sarkar S, Hopper AK, Hinnebusch AG (2000). Defects in tRNA processing and nuclear export induce GCN4 translation independently of phosphorylation of the alpha subunit of eukaryotic translation initiation factor 2.. Mol Cell Biol.

[23] McCormick MA, Delaney JR, Tsuchiya M, Tsuchiyama S, Shemorry A, Sim S, Chou AC, Ahmed U, Carr D, Murakami CJ, Schleit J, Sutphin GL, Wasko BM, Bennett CF, Wang AM, Olsen B, Beyer RP, Bammler TK, Prunkard D, Johnson SC, Pennypacker JK, An E, Anies A, Castanza AS, Choi E, Dang N, Enerio S, Fletcher M, Fox L, Goswami S (2015). A Comprehensive Analysis of Replicative Lifespan in 4,698 Single-Gene Deletion Strains Uncovers Conserved Mechanisms of Aging.. Cell Metab.

[24] Steffen KK, MacKay VL, Kerr EO, Tsuchiya M, Hu D, Fox LA, Dang N, Johnston ED, Oakes JA, Tchao BN, Pak DN, Fields S, Kennedy BK, Kaeberlein M (2008). Yeast life span extension by depletion of 60s ribosomal subunits is mediated by Gcn4.. Cell.

[25] Matecic M, Smith DL, Pan X, Maqani N, Bekiranov S, Boeke JD, Smith JS (2010). A microarray-based genetic screen for yeast chronological aging factors.. PLoS Genet.

[26] Labunskyy VM, Gerashchenko MV, Delaney JR, Kaya A, Kennedy BK, Kaeberlein M, Gladyshev VN (2014). Lifespan extension conferred by endoplasmic reticulum secretory pathway deficiency requires induction of the unfolded protein response.. PLoS Genet.

[27] Hughes AL, Hughes CE, Henderson KA, Yazvenko N, Gottschling DE (2016). Selective sorting and destruction of mitochondrial membrane proteins in aged yeast.. eLife.

[28] Ghavidel A, Baxi K, Ignatchenko V, Prusinkiewicz M, Arnason TG, Kislinger T, Carvalho CE, Harkness TA (2015). A Genome Scale Screen for Mutants with Delayed Exit from Mitosis: Ire1-Independent Induction of Autophagy Integrates ER Homeostasis into Mitotic Lifespan.. PLoS Genet.

[29] Gelino S, Hansen M (2012). Autophagy - An Emerging Anti-Aging Mechanism.. J Clin Exp Pathol.

[30] Madeo F, Zimmermann A, Maiuri MC, Kroemer G (2015). Essential role for autophagy in life span extension.. J Clin Invest.

[31] Kaur J, Debnath J (2015). Autophagy at the crossroads of catabolism and anabolism.. Nat Rev Mol Cell Biol.

[32] Tooze SA, Dikic I (2016). Autophagy Captures the Nobel Prize.. Cell.

[33] Netea-Maier RT, Plantinga TS, van de Veerdonk FL, Smit JW, Netea MG (2016). Modulation of inflammation by autophagy: Consequences for human disease.. Autophagy.

[34] Tyler JKaJ (2017). The role of autophagy in the regulation of yeast lifespan.. Ann N Y Acad Sci.

[35] Sinclair DA, Guarente L (1997). Extrachromosomal rDNA circles - a cause of aging in yeast.. Cell.

[36] Shcheprova Z, Baldi S, Frei SB, Gonnet G, Barral Y (2008). A mechanism for asymmetric segregation of age during yeast budding.. Nature.

[37] Aguilaniu H, Gustafsson L, Rigoulet M, Nystrom T (2003). Asymmetric inheritance of oxidatively damaged proteins during cytokinesis.. Science.

[38] Jazwinski SM (2004). Yeast replicative life span - the mitochondrial connection.. FEMS Yeast Res.

[39] Kaeberlein M, Powers 3rd RW, Steffen KK, Westman EA, Hu D, Dang N, Kerr EO, Kirkland KT, Fields S, Kennedy BK (2005). Regulation of yeast replicative life span by TOR and Sch9 in response to nutrients.. Science.

[40] Lin SJ, Defossez PA, Guarente L (2000). Requirement of NAD and SIR2 for life-span extension by calorie restriction in Saccharomyces cerevisiae.. Science.

[41] Talloczy Z, Jiang W, Virgin HWt, Leib DA, Scheuner D, Kaufman RJ, Eskelinen EL, Levine B (2002). Regulation of starvation- and virus-induced autophagy by the eIF2alpha kinase signaling pathway.. Proc Natl Acad Sci U S A.

[42] Ecker N, Mor A, Journo D, Abeliovich H (2010). Induction of autophagic flux by amino acid deprivation is distinct from nitrogen starvation-induced macroautophagy.. Autophagy.

[43] Abeliovich H, Dunn Jr WA, Kim J, Klionsky DJ (2000). Dissection of autophagosome biogenesis into distinct nucleation and expansion steps.. J Cell Biol.

[44] Nakatogawa H, Ichimura Y, Ohsumi Y (2007). Atg8, a ubiquitin-like protein required for autophagosome formation, mediates membrane tethering and hemifusion.. Cell.

[45] Bernard A, Jin M, Xu Z, Klionsky DJ (2015). A large-scale analysis of autophagy-related gene expression identifies new regulators of autophagy.. Autophagy.

[46] Jiang JC, Jaruga E, Repnevskaya MV, Jazwinski SM (2000). An intervention resembling caloric restriction prolongs life span and retards aging in yeast.. FASEB J.

[47] Kaya A, Lee BC, Gladyshev VN (2015). Regulation of protein function by reversible methionine oxidation and the role of selenoprotein MsrB1.. Antioxid Redox Signal.

[48] Kamada Y, Funakoshi T, Shintani T, Nagano K, Ohsumi M, Ohsumi Y (2000). Tor-mediated induction of autophagy via an Apg1 protein kinase complex.. J Cell Biol.

[49] Kamada Y, Yoshino K, Kondo C, Kawamata T, Oshiro N, Yonezawa K, Ohsumi Y (2010). Tor directly controls the Atg1 kinase complex to regulate autophagy.. Mol Cell Biol.

[50] Eisenberg T, Knauer H, Schauer A, Buttner S, Ruckenstuhl C, Carmona-Gutierrez D, Ring J, Schroeder S, Magnes C, Antonacci L, Fussi H, Deszcz L, Hartl R, Schraml E, Criollo A, Megalou E, Weiskopf D, Laun P, Heeren G, Breitenbach M, Grubeck-Loebenstein B, Herker E, Fahrenkrog B, Frohlich KU, Sinner F, Tavernarakis N, Minois N, Kroemer G, Madeo F (2009). Induction of autophagy by spermidine promotes longevity.. Nat Cell Biol.

[51] Speldewinde SH, Grant CM (2015). Spermidine cures yeast of prions.. Microb Cell.

[52] Teixeira MC, Cabrito TR, Hanif ZM, Vargas RC, Tenreiro S, Sa-Correia I (2011). Yeast response and tolerance to polyamine toxicity involving the drug : H+ antiporter Qdr3 and the transcription factors Yap1 and Gcn4.. Microbiology.

[53] Delaney JR, Ahmed U, Chou A, Sim S, Carr D, Murakami CJ, Schleit J, Sutphin GL, An EH, Castanza A, Fletcher M, Higgins S, Jelic M, Klum S, Muller B, Peng ZJ, Rai D, Ros V, Singh M, Wende HV, Kennedy BK, Kaeberlein M (2013). Stress profiling of longevity mutants identifies Afg3 as a mitochondrial determinant of cytoplasmic mRNA translation and aging.. Aging Cell.

[54] Heeren G, Rinnerthaler M, Laun P, von Seyerl P, Kossler S, Klinger H, Hager M, Bogengruber E, Jarolim S, Simon-Nobbe B, Schuller C, Carmona-Gutierrez D, Breitenbach-Koller L, Muck C, Jansen-Durr P, Criollo A, Kroemer G, Madeo F, Breitenbach M (2009). The mitochondrial ribosomal protein of the large subunit, Afo1p, determines cellular longevity through mitochondrial back-signaling via TOR1.. Aging.

[55] Holbrook MA, Menninger JR (2002). Erythromycin slows aging of Saccharomyces cerevisiae.. J Gerontol A Biol Sci Med Sci.

[56] Wu Z, Song L, Liu SQ, Huang D (2014). Tanshinones extend chronological lifespan in budding yeast Saccharomyces cerevisiae.. Appl Microbiol Biotechnol.

[57] Ables GP, Johnson JE (2017). Pleiotropic responses to methionine restriction.. Exp Gerontol.

[58] Wu Z, Song L, Liu SQ, Huang D (2013). Independent and additive effects of glutamic acid and methionine on yeast longevity.. PloS one.

[59] Johnson JE, Johnson FB (2014). Methionine restriction activates the retrograde response and confers both stress tolerance and lifespan extension to yeast, mouse and human cells.. PloS one.

[60] Alvers AL, Fishwick LK, Wood MS, Hu D, Chung HS, Dunn Jr WA, Aris JP (2009). Autophagy and amino acid homeostasis are required for chronological longevity in Saccharomyces cerevisiae.. Aging Cell.

[61] Pyo JO, Yoo SM, Ahn HH, Nah J, Hong SH, Kam TI, Jung S, Jung YK (2013). Overexpression of Atg5 in mice activates autophagy and extends lifespan.. Nature Commun.

